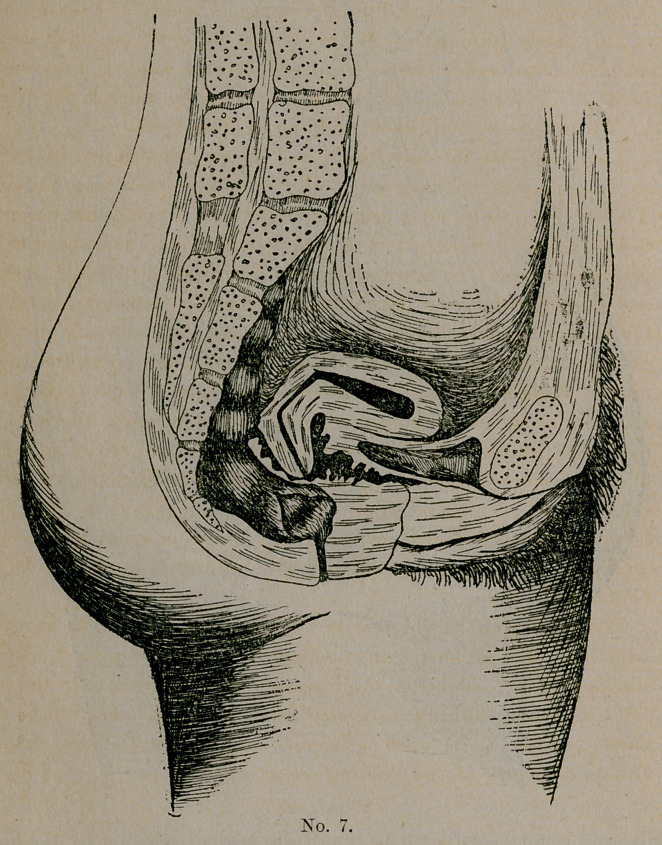# Ante and Retro-Positions of the Uterus—Their Pathology, Symptomatology and Treatment

**Published:** 1893-09

**Authors:** W. W. Stewart

**Affiliations:** Columbus, Ga.


### ANTE AND RETRO-POSITIONS OF THE UTERUS—THEIR PATHOLOGY, SYMPTOMATOLOGY AND TREATMENT.

By W. W. STEWART, M. D.
COLUMBUS, GA.

CASE 5.—Mrs. P., age thirty, four children, was sent to me from Florida, for bladder trouble and epileptic fits. At birth of last child, three years ago, had considerable trouble, the nature of which could not be learned. Was in labor forty-eight hours when an instrumental delivery was accomplished, after which she had fever for two weeks and was in bed forty-five days. On resuming her duties was troubled with a constant desire to pass water. Pain in loins; bad and almost constant headache, occipito-parietal. Constant leucorrhea, constipation to a fearful extent.

Two months after resuming her duties, patient was seized with what was diagnosed to be epilepsy of the grand mal type, for which she received constant treatment to no avail.

Attacks grew more frequent and more prolonged. After an attack would remain unconscious for six to seven hours.

This was her condition, augmented by irritated dyspepsia and dilated stomach, when I first saw her.

Uterus retroflexed and drawn backward and upward by sacro-uterine ligaments. Marked endometritis. Roof of bladder taut and paralyzed. Marked cystitis, 20 per cent. by measure of pus in urine.

Perineum torn to second degree which is not shown in this drawing. Cervix bilaterally lacerated. Facies uterina marked.

She was first curetted and uterus packed with iodoform gauze.

Six days thereafter cervix was operated upon and in fourteen days perineorraphy was performed. After this had no more epileptic seizures and returned home in good health, the uterus returning to normal position after curetting and other operations mentioned.

Our next case, is another form of retroversion, which for its causation has a contraction of the sacro-uterine ligaments, without other pathological conditions than those secondary to version.

Plate No. 5 represents an interesting case sent me from Eufaula, Alabama. Mrs. R., age twenty-five, had two children. With the first there was no trouble, patient being perfectly well till the birth of second child, two years ago, at which time she had some slight septic trouble which caused a rise of temperature for several days, which soon subsided. She had at this time some soreness over abdomen with tympanites. On coming to me her greatest complaint was from bladder in the form of chronic cystitis, and also obstinate constipation, with chronic dyspepsia and meteorism. On examination a clear diagnosis was made of anteversion, due to chronic posterior parametritis and metritis. The sacro-uterine ligaments were contracted, and cervix fixed. Bladder was examined with cystoscope and a large ulcer detected, caused by decomposition of urine in the bladder. Treatment consisted, first, in curetting and packing uterus with iodoform gauze. After four days this was withdrawn and a tenaculum caught in posterior lip of cervix, and gentle traction practiced for ten minutes at each sitting—they being every other day. The ligaments stretched quite rapidly, and with the aid of glycerine tampons and hot water in four weeks cervix was about in its normal position. The bladder trouble was treated at the same time, and at the end of two months patient was discharged with a cradle pessary, which I removed permanently five months after, to find everything in good condition and patient enjoying good health.

Plate No. 6 gives us an illustration of a form of anteflexion

No. 5.

often found after confinement when there has been some septic infection, followed, as is always true in such instances, by subinvolution. The fundus uteri becoming too heavy for its supports, falls either forward or backward, as the determining influence tends. In this instance forward. If this patient should fall into my hands soon after the deformity had taken place, I would hope—with good grounds—for a cure. I would first treat the metritis and endometritis, which is sure to exist, then the subinvolution, with the potash

No. 6.

salts and ergot, assisting it to retain the normal position by the aid of a cradle-shaped pessary and douches, which will aid largely in reducing its size. Add to this good tonics, an abdominal belt, skirt supports, corset waist, good food and fresh air, and our treatment is complete.

Plate No. 7. Gentlemen, this is a case which brings out a point in general medicine which it is well for us to constantly bear in mind. This represents the case of Mrs. S., age twenty-seven, who came to me from Macon. When seventeen years old, had a

No. 7.

protracted spell of typhoid fever. During the two months following her recovery she grew ten inches in height, was pale and anemic, and entire system was in a run-down condition. Being a young lady of fashion, she was put into corsets at once. Three months after her recovery, she menstruated for the first time after her illness. For two days prior, and all during its course, she suffered intense pain, bearing down in character. This dysmenorrhea continued, gradually increasing. Appetite became capricious, bowels constipated, and the long line of symptoms mentioned in the first of this paper developed. At twenty-two she married, after which all symptoms became worse until she became what I term a sofa invalid. In this condition she was treated by several physicians in Macon, one in Baltimore, and one in New York.

Then she came into my hands, and the results attained by us all so far are not at all flattering. Her condition now is as follows: The uterine syndrome is complete; abdomen, hyperesthetic; anteflexion of body of cervix with retro-position and dextro-rotation; bladder irritable; no cystitis; ovaries and tubes exquisitely tender and slightly enlarged. In the last year has developed amenorrheas but at each period, though flow consists only of about one drachm of bloody mucus, she suffers with a burning, boring pain in uterus, shooting pains and intense headaches. These headaches are more or less continuous during the entire menstrual period. I have both packed and dilated the uterus, and tried to use supports of every conceivable kind, but she can tolerate none of them. Cotton wool even gives pain. The second flexion is so high that cervical amputation or incision would be of no avail, and ovariotomy is my last resort, which I will perform on my return to Columbus.

The great point of interest in this case is a warning to us all to admonish our patients, convalescing from weakening or wasting diseases, or any condition diminishing muscular tone, to avoid corsets and heavy clothing suspended from the waist. Instead of the corset use the corset-waist. In this case we see the dire results from a disregard of the existing conditions.

(*Concluded.*)

## Figures and Tables

**No. 5. f1:**
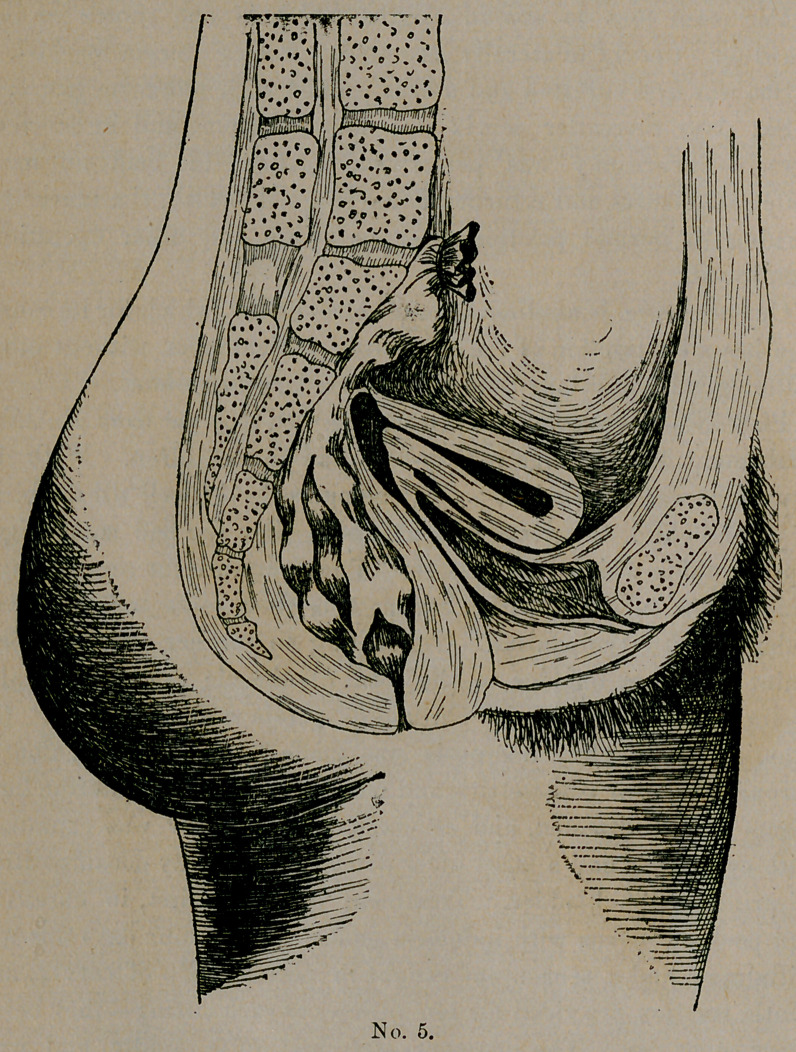


**No. 6. f2:**
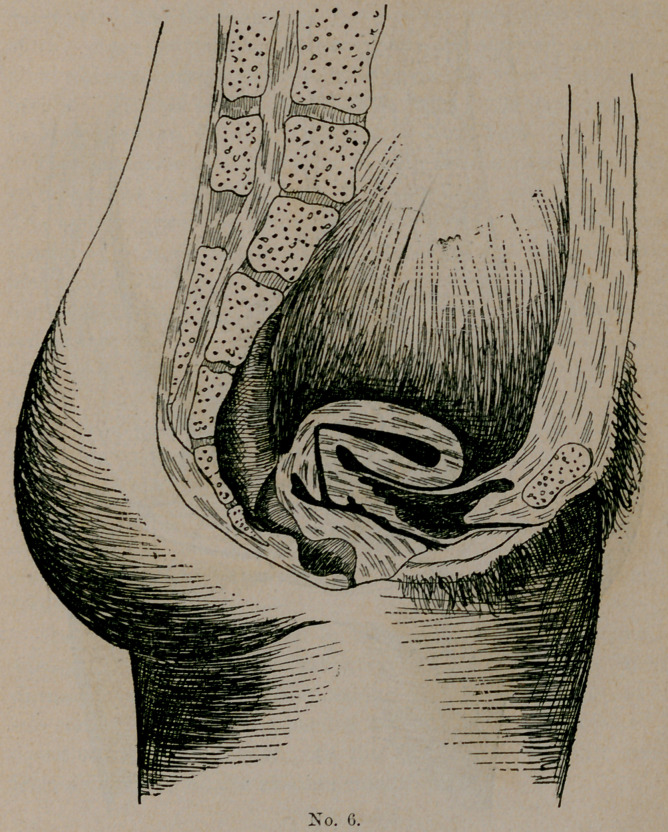


**No. 7. f3:**